# Stress to stability: Sense of coherence as a buffer against pandemic-related psychological distress

**DOI:** 10.4102/hsag.v30i0.2859

**Published:** 2025-06-24

**Authors:** Anita Padmanabhanunni, Tyrone B. Pretorius

**Affiliations:** 1 Department of Psychology, Faculty of Community and Health Science, University of the Western Cape, Cape Town, South Africa

**Keywords:** COVID-19, hopelessness, mental health, sense of coherence, set-point theory

## Abstract

**Background:**

Identifying protective factors in mental health-related outcomes is crucial, offering insights into the vulnerabilities and strengths individuals harness against psychological distress. There has been limited focus on exploring complex mediation and moderation models, which can uncover the relationships between stressors, protective factors and wellbeing.

**Aim:**

This study investigated the interrelationship between perceived stress, sense of coherence (SOC), and psychological distress.

**Setting:**

South African university students (*N* = 322) completed the Perceived Stress Scale, Sense of Coherence Scale-13, Beck Hopelessness Scale-9 and Center for Epidemiological Studies Depression Scale-10.

**Methods:**

Moderation analysis was conducted using the PROCESS macro to examine the role of SOC in moderating the relationship between perceived stress and psychological distress. Where moderation was not significant, mediation analysis was conducted.

**Results:**

Sense of coherence demonstrated multiple roles in mental health, exhibiting direct effects on indicators of psychological distress. Sense of coherence moderated the relationship between perceived stress and hopelessness. Under heightened stress conditions, individuals with low to medium SOC displayed more profound feelings of hopelessness compared to those with high SOC. Mediation analysis showed that SOC served as a bridge between perceived stress and both depression and anxiety. The identification of a potential SOC threshold offers a novel perspective on assessing risk levels, suggesting that individuals with low to moderate SOC are particularly vulnerable under high stress.

**Conclusion:**

The findings emphasise the need for targeted approaches to strengthen SOC as a resilience-enhancing factor.

**Contribution:**

The study advances theoretical discussions on stress-buffering models and offers guidance for mental health practitioners working in high-stress environments.

## Introduction

The quest to identify protective factors in mental health emerges from a critical need to understand the vulnerabilities and strengths that individuals possess to counteract psychological distress. While the study of risk factors offers insights into potential triggers and predispositions for mental health disorders, a singular focus on these aspects can potentially perpetuate a deficit-oriented perspective, overshadowing the inherent strengths, coping mechanisms and resilience that individuals exhibit in the face of adversity (Antonovsky 1987). Protective factors serve as a counterbalance, highlighting the capacities, resources and conditions that promote resilience, well-being and adaptability amid stressful events. They refer to those factors that can potentially influence, mitigate or modify an individual’s response to a stressor, thereby reducing the likelihood of an adverse mental health outcome (Antonovsky 1987).

Following this line of inquiry, researchers have delved into understanding the operational mechanisms of these protective factors, predominantly through direct and indirect pathways. The direct pathway posits that the protective factor itself directly influences mental health outcomes (Pretorius 2020). Conversely, the indirect pathway suggests that protective factors exert their influence by affecting other variables (e.g. stress appraisal, ego-resilience or perceptions of support), which in turn impact mental health outcomes. However, this bifurcated approach may oversimplify the multifaceted ways in which protective factors interact with various psychosocial elements (Kulcar et al. 2023). There has been less emphasis on examining more intricate mediation and moderation models, which might capture the interplay between protective factors, stressors and mental health outcomes (Kulcar et al. 2023). This study aims to contribute to the research in this area by examining the pathways through which sense of coherence (SOC) operates, both as a direct protective factor and as a potential mediator or moderator in the relationship between perceived stress and psychological distress.

The coronavirus disease 2019 (COVID-19) outbreak significantly increased perceived stress, anxiety, depression and hopelessness globally (Parvar et al. 2022; Stamatis et al. 2022). Perceived stress, the appraisal of stressors as uncontrollable or overwhelming, affects mental health (Cohen, Kamarck & Mermelstein 1983). Research during the outbreak confirmed the link between perceived stress and psychological distress. A study on Chinese adolescents found that those viewing pandemic stressors as more threatening experienced more depressive symptoms, but character strengths could mitigate these effects (Liu & Wang 2021). Similarly, Spanish nurses facing high perceived stress showed increased depression and anxiety symptoms, with resilience acting as a protective mental health factor (Lara-Cabrera et al. 2021). In Canada, individuals more inclined to self-isolate reported higher stress, depression and anxiety levels (Nkire et al. 2021). Moreover, a study on Mexican postpartum women found a higher prevalence of postpartum depression compared to North American counterparts, alongside elevated stress and anxiety levels, underscoring the pandemic’s broad mental health impacts (Suárez-Rico et al. 2021).

Existing studies have also underscored the pervasiveness of hopelessness during the disease outbreak (Goodwill 2023; Kasapoglu 2022). Hopelessness is conceptualised as a cognitive schema characterised by negative expectations about the future (Beck et al. 1974). A Turkish study found COVID-19-related anxiety linked to hopelessness, but self-efficacy and spirituality mitigated these feelings by fostering a sense of meaning in life (Kasapoglu 2022). Knowles and colleagues reported that hopelessness and resilience moderated the relationship between COVID-19-related stress and suicidal thoughts among a Welsh sample of adults (Knowles et al. 2022).

While the mental health impact of the COVID-19 pandemic is well established, existing studies have highlighted the salience of protective factors (Havnen et al. 2020; Kubo, Sugawara & Masuyama 2021) that can act as buffers, mitigating the adverse effects of stressful life events and promoting psychological health and well-being. The current study focusses on Sense of Coherence (SOC) as a potential protective resource (Antonovsky 1996). Sense of coherence is a global orientation or trait-like characteristic encompassing three central components – comprehensibility, manageability and meaningfulness. Comprehensibility entails the perception that life is structured, meaningful and predictable, while manageability entails the appraisal that one has the intrinsic capacities and external resources to cope effectively with life stressors. Meaningfulness refers to the perception that the challenges encountered in life are worth engaging in (Antonovsky 1996). Protective factors function by mitigating the impact of stressors, thereby reducing the likelihood of negative mental health outcomes. Sense of Coherence aligns with this definition, as it reflects an individual’s ability to interpret stressors in a way that fosters resilience. Specifically, comprehensibility, manageability and meaningfulness serve as mechanisms through which protective factors operate.

In the context of the pandemic, SOC can exert a direct influence on mental health outcomes and can serve as a salient protective factor (Padmanabhanunni 2022). A strong SOC can potentially provide a cognitive lens through which stressors are appraised as comprehensible, manageable and meaningful. Several studies across different cultures and contexts have demonstrated the protective effects of a strong SOC against adverse mental health outcomes (George-Levi et al. 2020; Padmanabhanunni 2022; Veronese, Mahamid & Bdier 2022).

A sense of coherence can also influence mental health through mediating and moderating pathways. Mediating pathways involve SOC acting as a mechanism through which external stressors translate to mental health outcomes (Pretorius 2020). On the other hand, moderating pathways imply that SOC can change the strength or direction of the relationship between a stressor and mental health (Pretorius 2020). In this role, SOC acts as a variable that conditions the effect of a stressor. By understanding these pathways towards coping, it is possible to gain a nuanced perspective of the dynamic interaction of SOC with stressors and its role in shaping mental health outcomes. The current study is grounded in the stress-buffering hypothesis, which proposes that protective factors such as SOC interact with stressors to determine mental health outcomes (Antonovsky 1996).

## Research methods and design

### Setting

The study design was cross-sectional and conducted at a South African higher education institution in the Western Cape province. Data were collected during March–August 2022, which coincided with the fifth wave of the pandemic in South Africa with a rapid spread of cases because of the BA.5.2.1.7 variant (Vollgraaff 2022).

### Participants and procedures

The study participants were university students enrolled at a higher education institution. The recruitment process involved distributing detailed information regarding the study to the student body through the Registrar’s office. This communication included a brief description of the study and its aims and an invitation to participate by accessing an electronic survey via a provided link. The survey was hosted on Google Forms, which facilitated the online administration of the various instruments detailed in the Measures section of this study. A total of 322 completed questionnaires were received, yielding a response rate of 18.9%. The majority of the sample resided in an urban area (87.3%) and were women (77%). The mean age of the sample was 26.01 years (s.d. [standard deviation] = 10.19) (Pretorius & Padmanabhanunni 2023b). Most participants (86.6%) reported being vaccinated, and 25.5% had contracted the virus. Close to half the sample (40.7%) reported having lost a family member because of COVID-19 infection.

### Measures

#### Perceived Stress Scale

The Perceived Stress Scale [(PSS) (Cohen 1988)] measures how much individuals find life events stressful, using a 10-item, five-point scale from ‘never’ (0) to ‘very often’ (4). Higher scores indicate greater perceived stress. The scale’s reliability and validity were confirmed with a 0.78 reliability coefficient and its correlation with life events and stress measures. It has been effectively used in South Africa, where Nekgotha et al. (2020) reported a reliability of 0.73 in a university student sample.

#### Sense of Coherence Scale

Sense of coherence was measured using the Sense of Coherence Scale-13 [(SOCS-13) (Antonovsky 1993)]. It is scored on a seven-point scale with different scale anchors for the various items. Higher scores on the SOCS-13 indicate that participants regard events in their lives as manageable, meaningful and comprehensible. In South Africa, the SOCS-13 has been used with a teacher (Pretorius & Padmanabhanunni 2022) as well as a student sample (Padmanabhanunni & Pretorius 2022), and both studies reported a reliability coefficient of 0.81.

#### Beck Hopelessness Scale

The indices of psychological distress that were measured in the current study included hopelessness, depression and anxiety. The authors used the nine-item version (Balsamo et al. 2020) of the original 20-item Beck Hopelessness Scale [(BHS) (Beck et al. 1974)] to assess hopelessness. The BHS consists of nine items that are scored on a dichotomous scale of true or false. High scores reflect a pessimistic outlook on life and a sense of hopelessness. To the authors’ knowledge, the nine-item version of the BHS has not been previously used in South Africa.

#### Center for Epidemiological Studies Depression Scale

Depression was assessed using the 10-item version (Andresen et al. 1994) of the original 20-item Center for Epidemiological Studies Depression Scale [(CES-D) (Radloff 1977)]. The CES-D10 consists of 10 items that are scored on a four-point scale that ranges from *rarely or none of the time* to *most or all of the time*. Higher scores on the CES-D10 reflect higher levels of depression. Andresen and colleagues reported a test-retest reliability of 0.71 in a sample of older adults in the relationship between scores on the CES-D10 and health status scores as well as positive affect, providing evidence of the validity of the CES-D10 (Andresen et al. 1994). Baron, Davies and Lund (2017) validated the CES-D10 in different language groups in South Africa and reported reliability coefficients ranging between 0.69 and 0.89.

#### State-Trait Anxiety Inventory

Anxiety was measured with the five-item version of the trait scale of the State-Trait Anxiety Inventory [(STAI-T5) (Zsido et al. 2020)], which is a shortened version of the original 20-item STAI-T5 (Spielberger 1985). Responses to the items of the STAI-T5 are made on a four-point scale that ranges from *not at all* (1) to *very much so* (4). Higher scores on the STAI-T5 reflect higher levels of anxiety. The developers reported an alpha coefficient of 0.86 for the STAI-T5. A South African study used both item response theory and classical test theory and reported satisfactory reliability (α = 0.89, Mokken scale reliability = 0.89) and confirmed that the STAI-T5 is a unidimensional measure of anxiety (Pretorius & Padmanabhanunni 2023a).

### Data analysis

For all analyses, IBM SPSS for Windows version 28 (IBM Corp., Armonk, NY, US) was used. The authors obtained descriptive statistics (means and standard deviations), intercorrelations between variables (Pearson *r*) and indices of reliability (alpha and omega) for all scales. They examined gender differences using two-sample *t*-tests as well as the relationship between age and all the study variables (Pearson *r*) to determine whether age and gender are potential confounders.

In line with this study’s theoretical framework, the authors utilised the PROCESS macro in SPSS (Hayes 2017) to explore how SOC moderates the impact of perceived stress on hopelessness. This involved creating an interaction term from the predictor (perceived stress) and moderator (SOC), with both variables mean centred to reduce multicollinearity. The significance of this term was assessed with 95% bootstrapped confidence intervals. The authors analysed the moderation by plotting regression slopes at three SOC levels: low, medium and high.

Where there was a significant interaction effect, the exact score on the moderator where the relationship between the predictor and the outcome variable ceased to be significant was determined using the Johnson-Neyman technique (Johnson & Neyman 1936).

### Ethical considerations

Ethical approval to conduct the study was obtained from the Humanities and Social Sciences Ethics Committee of the University of the Western Cape (reference no.: HS22/2/9). Participants provided written informed consent, participation was voluntary and anonymous.

## Results

The indices of skewness and kurtosis, descriptive statistics reliability of scales and intercorrelations between study variables are reported in Table 1.

**TABLE 1:** Descriptive statistics, reliabilities and intercorrelations between study variables.

Variables	1	2	3	4	5	6
1. Age	-	-	-	-	-	-
2. Perceived stress	−0.32**	-	-	-	-	-
3. Sense of coherence	0.34**	−0.65**	-	-	-	-
4. Hopelessness	−0.17*	0.47**	−0.53**	-	-	-
5. Depression	−0.25**	0.66**	−0.69**	0.50**	-	-
6. Anxiety	−0.35**	0.60**	−0.63**	0.46**	0.66**	-
Mean	26.01	23.89	47.63	2.29	14.15	12.36
s.d.	10.19	6.28	12.92	2.45	6.77	4.13
Skewness	1.76	−0.18	0.23	1.21	0.05	0.03
Kurtosis	2.45	−0.18	−0.16	0.59	−0.73	−0.88
Alpha	-	0.85	0.83	0.84	0.84	0.88
Omega	-	0.86	0.84	0.84	0.85	0.88

s.d., standard deviation.

* , *p* < 0.01;

** , *p* < 0.001.

Table 1 shows that the skewness values (−0.18 to 1.76) and the kurtosis values (−0.16 to 2.45) were all within an acceptable range, indicating that the data were approximately normally distributed. The reliability of all the scales can be considered satisfactory (α = 0.83–0.88; ω = 0.84–0.88). Age was significantly negatively associated with perceived stress (*r* = −0.32, *p* < 0.001, medium effect), hopelessness (*r* = −0.17, *p* < 0.001, small effect), depression (*r* = −0.25, *p* < 0.001, effect) and anxiety (*r* = −0.35, *p* < 0.001, medium effect) and significantly positively associated with SOC (*r* = 0.34, *p* < 0.001, medium effect). Older age was thus associated with higher levels of SOC and lower levels of perceived stress, hopelessness, depression and anxiety.

Perceived stress was significantly negatively associated with SOC (*r* = −0.65, *p* < 0.001, large effect) and significantly positively associated with hopelessness (*r* = 0.47, *p* < 0.001, medium effect), depression (*r* = 0.66, *p* < 0.001, large effect) and anxiety (*r* = 0.60, *p* < 0.001, large effect). Higher levels of perceived stress were thus associated with higher levels of hopelessness, depression and anxiety and lower levels of SOC.

Sense of coherence was significantly negatively associated with hopelessness (*r* = −0.53, *p* < 0.001, large effect), depression (*r* = −0.69, *p* < 0.001, large effect) and anxiety (*r* = −0.63, *p* < 0.001, large effect). Thus, higher levels of SOC were associated with lower levels of hopelessness, depression and anxiety.

With respect to gender differences, two sample *t*-tests indicated that there were only gender differences in terms of SOC and not for any of the other variables. In particular, it was found that men reported higher levels of SOC ($\bar{X}$ = 50.45, s.d. = 13.03) than women ($\bar{X}$ = 46.79, s.d. = 12.79, *t* = 2.15, *p* = 0.03). Given the gender differences and the relationship of age with all the variables, gender and age were added as covariates in the further analysis.

The results of the moderation analysis with perceived stress as a predictor, SOC as a moderator and the indices of psychological distress as the dependent variables are reported in Table 2.

**TABLE 2:** The moderating role of sense of coherence in the perceived stress-psychological distress relationship.

Variable	*β*	s.e.	95% CI	*p*-value
**Anxiety as dependent variable**				
Perceived stress	0.201	0.038	0.132, 0.272	< 0.001
Sense of coherence	−0.126	0.017	−0.160, -0.091	< 0.001
Sense of coherence X perceived stress	0.002	0.002	−0.002, 0.005	0.295
**Depression as dependent variable**				
Perceived stress	0.405	0.054	0.298, 0.511	< 0.001
Sense of coherence	−0.240	0.026	−0.291, -0.188	< 0.001
Sense of coherence X perceived stress	−0.003	0.002	−0.008, 0.003	0.321
**Hopelessness as dependent variable**				
Perceived stress	0.102	0.023	0.056, 0.147	< 0.001
Sense of coherence	−0.075	0.011	−0.098, -0.053	< 0.001
Sense of coherence X perceived stress	−0.006	0.001	−0.009, -0.004	< 0.001

s.e., standard error; CI, confidence intervals; *β*, beta.

**FIGURE 1:**
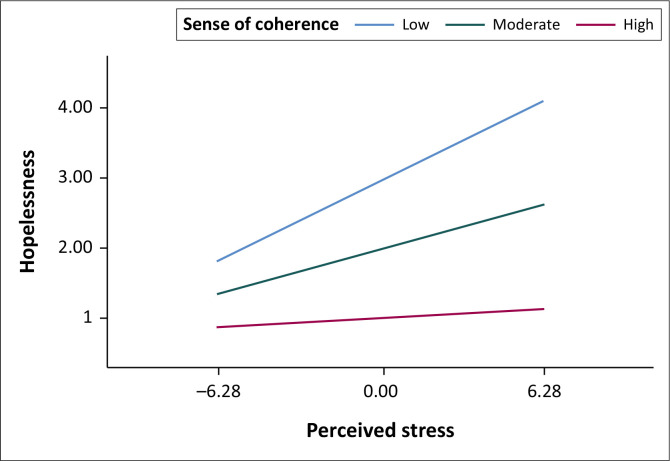
Plot of the interaction between the sense of coherence and perceived stress in the relationship with hopelessness.

Table 2 indicates that the moderating effect (SOC X perceived stress) was only significant for the relationship between perceived stress and SOC (*β* = −0.006, *p* < 0.001) but not for the relationships between perceived stress and anxiety (*β* = 0.002, *p* = 0.295) or depression coherence (*β* = −0.003, *p* = 0.321). The nature of the significant interaction in the case of hopelessness is plotted in Figure 1 for three different values of SOC.

Figure 1 demonstrates that the regression line at low and medium levels of SOC was much steeper than the regression line at high levels of SOC. At both low and high levels of perceived stress, the levels of hopelessness were higher for low and medium SOC than the levels of hopelessness for high SOC. Table 3 reports the statistical significance of the association between perceived stress and hopelessness at different levels of SOC.

**TABLE 3:** The association between perceived stress and hopelessness at different levels of sense of coherence.

Levels of sense of coherence	*β*	s.e.	95% CI	*p*-value
Low	0.182	0.029	0.125, 0.240	< 0.001
Medium	0.102	0.023	0.056, 0.147	< 0.001
High	0.021	0.026	−0.031, 0.073	0.430

s.e., standard error; CI, confidence intervals; *β*, beta.

Table 3 indicates that at low (*β* = 0.182, *p* < 0.001) and medium levels (*β* = 0.102, *p* < 0.001) of SOC, the relationship between perceived stress and hopelessness was direct and statistically significant, while it was nonsignificant at high levels of SOC (*β* = 0.021, *p* = 0.430).

**FIGURE 2:**
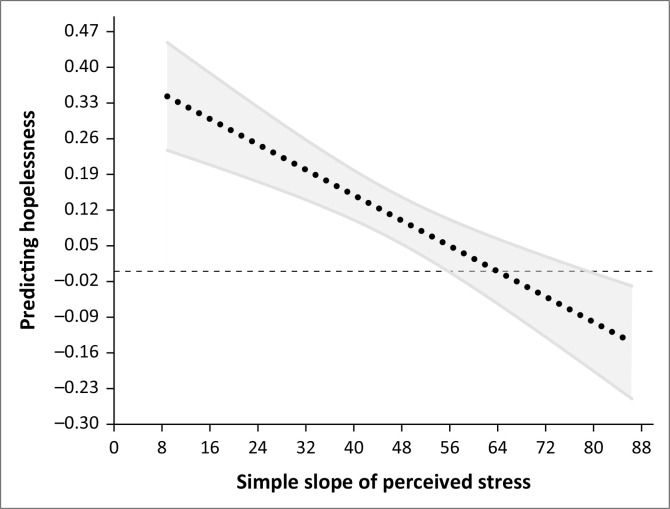
The Johnson-Neyman plot of the relationship between perceived stress and hopelessness for a range of sense of coherence values.

In Figure 1, the regression lines are plotted using arbitrarily selected low, medium and high values of the moderator. For example, the PROCESS macro also provides the option of the 16th, 50th and 84th percentile as potential values. Thus, these arbitrary values only provide information about the relationship between the predictor and the dependent variable at those particular scores. Therefore, the authors applied the Johnson-Neyman technique to plot the slope across a range of SOC values, identifying the exact point at which the relationship between perceived stress and hopelessness ceases to be significant. The Johnson-Neyman plot is reported in Figure 2.

In Figure 2, the grey area represents the confidence interval for the effect of perceived stress on hopelessness. The point at which the confidence interval includes zero (i.e. the crossover where the relationship between perceived stress and hopelessness ceases to be significant) is 55.73, as indicated by the first vertical line. There is another crossover at 79.12. However, the maximum value of SOC in the current study was 80, and it is recommended that such extreme values should not be considered (Carden, Holtzman & Strube 2017).

The results of the mediation analysis with perceived stress as a predictor, depression and hopelessness as dependent variables and SOC as a mediator are presented in Table 4.

**TABLE 4:** The mediating role of sense of coherence in the relationship between perceived stress and depression as well as anxiety.

Effects	*β*	s.e.	95%CI	*β*
**Direct effects**				
Sense of coherence → Depression	−0.240	0.026	−0.292, -0.188	−0.458
Sense of coherence → Anxiety	−0.126	0.017	−0.160, -0.091	−0.393
**Indirect effects**				
Perceived stress → Sense of coherence → Depression	0.297	0.042	0.219, 0.387	0.276
Perceived stress → Sense of coherence → Anxiety	0.155	0.027	0.105, 0.210	0.236

s.e., standard error; CI, confidence intervals; *β*, beta.

**FIGURE 3:**
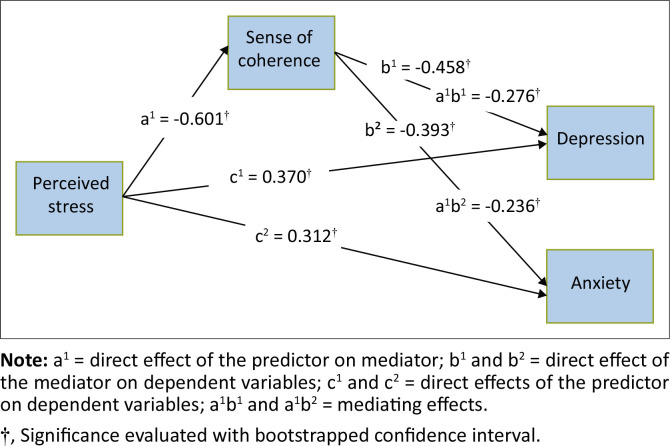
The mediating role of sense of coherence.

Table 4 indicates that SOC had a significant direct effect on depression (*β* = −0.458, [−0.292, −0.188]) and anxiety (*β* = −0.393, [−0.160, −0.091]). In addition, SOC mediated the relationship between perceived stress and depression (*β* = 0.276, [0.219, 0.387]) as well as anxiety (*β* = 0.236, [0.105, 0.210]). The mediating role of SOC is visually presented in Figure 3.

## Discussion

This study investigated the interrelationship between perceived stress, SOC and indices of psychological distress. Several key findings emerged from the study. Firstly, the study confirmed that SOC had a direct effect on depression, hopelessness and anxiety. Depression is characterised by a negative view of the self as flawed or deficient, appraisals of stressors as insurmountable obstacles and of the future as bleak (Beck & Haigh 2014). A stronger SOC can potentially counterbalance these negative appraisals by facilitating the reframing of challenges as potential opportunities for growth and propelling the individual to activate appropriate resources for coping (Del-Pino-Casado et al. 2019). In the case of anxiety, which often revolves around excessive worry about future uncertainties, a strong SOC can anchor individuals in a more grounded perspective, emphasising their capacities to manage and find coherence even in uncertain situations (Leung et al. 2022).

The findings are supported by the literature. Aderhold et al. (2019) found that SOC was the strongest negative predictor of depressive symptoms and distress among cancer patients. They proposed that cancer patients with higher levels of SOC may be better able to view their ill health as foreseeable and their cancer treatment as comprehensible. This belief may enhance their confidence in having the personal resources to cope, thereby mitigating distress. Dymecka, Gerymski and Machnik-Czerwik (2022) reported that greater levels of SOC had a negative association with fear of COVID-19 and proposed that this was because of the influence of SOC on individuals’ perceptions of their capacity to cope with stressors and access resources.

Secondly, SOC mediated the association between perceived stress and depression anxiety, respectively. This means that while perceived stress has the potential to precipitate and exacerbate feelings of depression and anxiety, this relationship is potentially shaped, channelled and often mitigated by the strength and presence of SOC. Individuals with a strong SOC may be better equipped to appraise perceived stressors in ways that are more adaptive and constructive. Rohani et al. (2015) found that SOC mediated the relationship between dimensions of health-related quality of life among patients with breast cancer, Einav and Margalit (2020) reported that SOC moderated the relationship between loneliness and hope among bereaved parents.

Thirdly, the study underscored the moderating role of SOC in the relationship between perceived stress and feelings of hopelessness. Differential impacts were observed based on the strength or level of SOC. For those with low to medium levels of SOC, the relationship between perceived stress and hopelessness was both direct and robust. In contrast, at elevated SOC values, the association between perceived stress and hopelessness was attenuated to the point of being statistically nonsignificant. This suggests that when SOC reaches a certain threshold, it acts as a substantial buffer, dampening the negative psychological repercussions of perceived stress. The Johnson-Neyman plot indicated a precise cutoff for this effect. At an SOC value of 55.73, the detrimental relationship between perceived stress and hopelessness ceased to be statistically significant. This suggests that SOC not only serves as a defence against psychological distress but also delineates a clear tipping point beyond, which its protective effects become particularly pronounced.

This study has particular implications. By confirming that SOC both mediates and moderates the relationships between perceived stress and mental health outcomes, the findings challenge the simplistic bifurcation of these pathways. The finding that a specific SOC value acts as a threshold for hopelessness amidst perceived stress can be integrated into the set-point theory of well-being. Researchers (Cummins 2015; Lucas 2007) have proposed that a set of psychological processes actively controls and maintains subjective well-being in a manner similar to the homeostatic maintenance of body temperature. The findings of this study suggest the possibility of an SOC set-point beyond which the protective effects become especially pronounced. Clinicians can potentially use this as a benchmark in risk assessments.

This study has certain limitations. The cross-sectional design allows for capturing data at a single point in time, which limits this study’s ability to infer causal relationships between the variables. Longitudinal studies would be needed to determine the directionality of the associations and to observe how these relationships evolve over time. The study relied on self-report measures, which might introduce social desirability and recall bias. The findings might be specific to the context of a global health crisis and might differ under normal circumstances. The study involved a population subgroup, and the findings may not be extrapolated to broader or different populations with confidence.

## Conclusion

This study underscores the central role of SOC in mediating and moderating the effects of perceived stress on indicators of psychological distress, namely depression, anxiety and hopelessness. The findings suggest that enhancing SOC can potentially serve as a therapeutic target, acting as a buffer against the adverse impacts of overwhelming stressors, especially during unprecedented times such as a global pandemic.
